# Multicriteria Optimization Model for the Study of the Efficacy of Skin Antiaging Therapy

**DOI:** 10.1155/2012/103919

**Published:** 2012-02-28

**Authors:** Maria Crişan, Guido Cappare, Luciana Neamţiu, Ioana Chiorean, Liana Lupşa, Diana Crişan, Radu Badea

**Affiliations:** ^1^Department of Histology, Iuliu Haţieganu University of Medicine and Pharmacy, Cluj-Napoca, Romania; ^2^Department of Aestetics, AMIA, Milano, Italy; ^3^Ion Chiricuţă Cancer Institute, Cluj-Napoca, Romania; ^4^Faculty of Mathematics and Computer Science, Babeş-Bolyai University, Cluj-Napoca, Romania; ^5^Iuliu Haţieganu University of Medicine and Pharmacy, Cluj-Napoca, Romania; ^6^Department of Ultrasonography, Iuliu Haţieganu University of Medicine and Pharmacy, Cluj-Napoca, Romania

## Abstract

The evolution of the cutaneous structure after topical treatment with P63 antiaging complex, assessed with high frequency ultrasound, is studied by means of multicriteria optimization model. Due to the fact that the impact of the treatment may influence the quality of life, a medical index which measures, from this point of view, the efficacy of the treatment is given, also taking into account medical and economical aspects.

## 1. Introduction

The basic idea of Pharmaco-Economics studies is to gain a physical and psychical comfort state for as long as possible, with the smallest amount of money. Therefore, according to these studies (see [1]) which consider a treatment in terms of results related to costs, one of the following five types of analysis is used: and cost-effects (CEA), cost-minimization (CMA), cost-utility (CUA), cost-efficiency (CEAC), and cost-benefit (CBA). Then, the data obtained after each of this analysis are used to compare two or more treatments. Cost-utility analysis was developed to help decision makers compare the value of alternative interventions that have very different health benefits, and it facilitates these comparisons without recourse to placing monetary values on different health states. The primary outcome of a cost-utility analysis is the cost per quality-adjusted life years (QALYs) or incremental cost-effectiveness ratio (ICER), which is calculated as the difference in the expected cost of two interventions, divided by the difference in the expected QALYs produced by the two interventions. QALYs measure health as a combination of the duration of life and the health-related quality of life. Also, there is another index, denoted by NB or INB, which means *incremental net benefit*, defined by

(1) $$\text{INB}=\lambda \Delta_{e}-\Delta_{c},$$

where *λ* is the willingness to pay. 

These indexes are largely used in the literature (see, e.g., [2–5]). Unfortunately, they may not always reflect with sufficient accuracy all aspects of medical outcomes of treatment, perception and impact of treatment on the patient's psyche, economic effects, and so forth.

Using the multicriteria optimization technique, in [6] a new index, called medicoeconomic index of a treatment (denoted by MEI), is introduced. Its construction may use all the desired aspects. It permits a simultaneous comparison of two or more medical treatments. And, in addition, due to the fact that it emphasizes the importance of every aspect in a general context, it gains an increased flexibility (see [7–10]).

There are papers in the literature which use multifactorial decisions to compare the medical treatments (see the book [11] and the studies [12, 13]).

The purpose of this paper is to show how the MEI index may best quantify the effect of applying a skin rejuvenation treatment, taking into account multiple aspects: changes in skin parameters occurred after treatment application, side effects, how the patient perceives the treatment and outcome of application, cost of the treatment.

Quantification, materialized by building the MEI index, permits comparisons among several types of treatments or comparing the results obtained with the same treatment applied to different groups of patients, groups that differ either by age or by phototype of the patients.

## 2. The Model on a Specific Sample

Let *T* be a medical treatment, applied against skin aging, whose effect we want to quantify. In particular we considered treatment with P63 antiaging complex.

From the treatment point of view, the patients were classified in two groups of study: the first group included persons to whom the new medication to be tested was applied, and the second group, the control group, to whom the old medication was given. It is important to mention that both groups were homogenous relatively to patient age, health state, and phenotype. From medical point of view, the obtained results refer to thickness of the epidermis (mm), thickness of the dermis (mm), number of low echogenity pixels (LEP), medium and high echogenity pixels (MEP and HEP), and the LEPs/LEPi ratio (low echogenic pixels in the upper dermis/low echogenic pixels in the lower dermis). All these imagistic parameters quantify both the skin aging process and the efficacy of the antiaging treatments. For each of these parameters, the mean values for every study group, age group, and phenotype group are given.

Taking this into account, for each treatment *T*_*i*_, *i* ∈ {1,2}, each age group *j*, *j* ∈ {1,2, 3}, and each phenotype *h*, *h* ∈ {2,3}, we indicate the following:

- *E*_*ijh*1_, *E*_*ijh*2_, the mean values of the thickness of epidermis of the patients who take the treatment *i*, are in age group *j* and have phenotype *h*, before the treatment, and, respectively, after the treatment;
- *D*_*ijh*1_, *D*_*ijh*2_, the mean values of the thickness of dermis of the patients who take the treatment *i*, are in age group *j* and have phototype *h*, before the treatment, respectively, after the treatment;
- *L*_*ijh*1_, *L*_*ijh*2_, the mean values of the LEP of the patients who take the treatment *i*, are in age group *j* and have phototype *h*, before the treatment, respectively after the treatment;
- *M*_*ijh*1_, *M*_*ijh*2_, the mean values of the MEP of the patients who take the treatment *i*, are in age group *j* and have phototype *h*, before the treatment, respectively, after the treatment;
-   
*H*_*ijh*1_, *H*_*ijk*2_, the mean values of the HEP of the patients who take the treatment *i*, are in age group *j* and have phototype *h*, before the treatment, respectively, after the treatment;
- *F*_*ijh*1_, *F*_*ijk*2_, the mean values of LEPi/LEPu;
- *R*_*ijh*_%, the percent of those with no adverse effects (erythema, pruritus, ocular disturbance, etc.);
- *C*_*ijh*_, the mean value of the cost of treatment for the period of time when the study was performed, taking into account a person in group *i*, of age *j* and a phototype *h*.

The patients were asked to fill in a questionnaire and give two marks, denoted by *q* and *s* ∈ {1,…, 10}, concerning the convenience of treatment application and the consequential effect of the treatment, respectively. We denote by *Q*_*ijh*_ and *S*_*ijk*_, respectively, the mean values of the marks given by the patients in age group *j* for the treatment *i*, and which have phototype *h*.

Let *K* be the maximum accepted value for the cost of the treatment for one patient during the study period of time.

### 2.1. Methods

The method used for comparison was that of introducing a medicoeconomic index, by means of multicriteria optimization, which permits a rigorous observation of the evolution of our patients.

For a protocol treatment *T*_*i*_, the percentage *P*_*ijh*_% of patients from treatment group *i*, age group *j*, and phototype *h*, is known.

Our problem was the following: having the previous information and taking into account the cost of the new treatment and the opinions of the patients, we wanted to determine the efficacy of the new treatment, and consequently, if it was worth using it. The expected medical results are increase of the mean thickness of epidermis and dermis, decrease of the mean number of low echogenity pixels, the increase of medium and high echogenity pixels, and the increase of LEPs/LEPi ratio. From economical point of view, it is desirable that the mean value of the cost for the new treatment to be as low as possible.

Finally, from the patients' point of view, the effect of the treatment is expected to be maximum possible and its application as comfortable as it can be.

In order to solve this problem, we compared the treatments at a general level (group 1 versus group 2 of treatments), but also on each age group and phenotype group. In every case, we had to solve a mathematical multicriteria optimization problem.

We introduced the variables *x*_1_ and *x*_2_, which take only the values 0 and 1. We have: *x*_1_ = 1, if the first treatment protocol is chosen, *x*_1_ = 0, if not; *x*_2_ = 1, if the second treatment protocol is chosen, *x*_2_ = 0, if not. Due to the fact that one patient has to follow one and only one treatment protocol, we have the obvious condition *x*_1_ + *x*_2_ = 1.

Then we consider the functions: *f*_*k*_ : {0,1}^2^→**R**, *k* ∈ {1,…, 60}, given for every *j* ∈ {1,2, 3} and *h* ∈ {2,3}, respectively, by

(2) $$\begin{array}{c} f_{2(j-1)+(h-1)}\left(x_{1},x_{2}\right)=P_{1jh}\left(\frac{E_{1jh2}}{E_{1jh1}}\right)x_{1}+P_{2jh}\left(\frac{E_{2jh2}}{E_{2jh1}}\right)x_{2},\;\forall \left(x_{1},x_{2}\right)\in {\left\{0,1\right\}}^{2},\\ f_{6+2(j-1)+(h-1)}\left(x_{1},x_{2}\right)=P_{1jh}\left(\frac{D_{1jh2}}{D_{1jh1}}\right)x_{1}+P_{2jh}\left(\frac{D_{2jh2}}{D_{2jh1}}\right)x_{2},\;\forall \left(x_{1},x_{2}\right)\in {\left\{0,1\right\}}^{2},\\ f_{12+2(j-1)+(h-1)}\left(x_{1},x_{2}\right)=P_{1jh}\left(\frac{L_{1jh1}}{L_{1jh2}}\right)x_{1}+P_{2jh}\left(\frac{L_{2jh1}}{L_{2jh2}}\right)x_{2},\;\forall \left(x_{1},x_{2}\right)\in {\left\{0,1\right\}}^{2},\\ f_{18+2(j-1)+(h-1)}\left(x_{1},x_{2}\right)=P_{1jh}\left(\frac{M_{1jh2}}{M_{1jh1}}\right)x_{1}+P_{2jh}\left(\frac{M_{2jh2}}{M_{2jh1}}\right)x_{2},\;\forall \left(x_{1},x_{2}\right)\in {\left\{0,1\right\}}^{2},\\ f_{24+2(j-1)+(h-1)}\left(x_{1},x_{2}\right)=P_{1jh}\left(\frac{H_{1jh2}}{H_{1jh1}}\right)x_{1}+P_{2jh}\left(\frac{H_{2jh2}}{H_{2jh1}}\right)x_{2},\;\forall \left(x_{1},x_{2}\right)\in {\left\{0,1\right\}}^{2},\\ f_{30+2(j-1)+(h-1)}\left(x_{1},x_{2}\right)=P_{1jh}\left(\frac{F_{1jh2}}{F_{1jh1}}\right)x_{1}+P_{2jh}\left(\frac{F_{2jh2}}{F_{2jh1}}\right)x_{2},\;\forall \left(x_{1},x_{2}\right)\in {\left\{0,1\right\}}^{2},\\ f_{36+2(j-1)+(h-1)}\left(x_{1},x_{2}\right)=-\left(\frac{R_{1jh}}{P_{1jh}}\right)x_{1}-\left(\frac{R_{2jh2}}{P_{2jh}}\right)x_{2},\;\forall \left(x_{1},x_{2}\right)\in {\left\{0,1\right\}}^{2},\\ f_{42+2(j-1)+(h-1)}\left(x_{1},x_{2}\right)=P_{1jh}\left(\frac{Q_{1jh}}{10}\right)x_{1}+P_{2jh}\left(\frac{Q_{2jh}}{10}\right)x_{2},\;\forall \left(x_{1},x_{2}\right)\in {\left\{0,1\right\}}^{2},\\ f_{48+2(j-1)+(h-1)}\left(x_{1},x_{2}\right)=P_{1jh}\left(\frac{S_{1jh}}{10}\right)x_{1}+P_{2jh}\left(\frac{S_{2jh}}{10}\right)x_{2},\;\forall \left(x_{1},x_{2}\right)\in {\left\{0,1\right\}}^{2},\\ f_{54+2(j-1)+(h-1)}\left(x_{1},x_{2}\right)=-P_{1jh}\left(\frac{C_{1jh}}{K}\right)x_{1}-P_{2jh}\left(\frac{C_{2jh}}{K}\right)x_{2},\;\forall \left(x_{1},x_{2}\right)\in {\left\{0,1\right\}}^{2}. \end{array}$$

Obviously, our purpose is to determine, simultaneously, their maximum. Generally, this fact cannot be fulfilled; therefore, a compromised solution is to be accepted: a synthesis function is considered and each of the previous functions gets a weight, according to the importance of every criterion in the problem.

So, firstly we attribute weights to the main elements of our final purpose:

- getting the best medical results (purpose indicated by the functions *f*_1_,…, *f*_36_, *f*_49_,…, *f*_54_);
- the best reception of the patient (functions *f*_43_,…, *f*_54_);
- the lowest possible cost of the treatment (indicated by the functions *f*_55_,…, *f*_60_)_._

Each of these purposes gets a nonnegative weight denoted by *t*_*u*_  (*u* ∈ {1,2, 3}), with the property that *t*_1_ + *t*_2_ + *t*_3_ = 1. Obviously, the three weights may be or may be not equal.

**Remark 1**

In some other cases, we may distinguish these weights even further. More precisely, the weight *t*_2_, corresponding to the way in which the patient is contained with the treatment may be considered as the sum between two others weights: *t*_*Q*_, for the convenience of treatment application and *t*_*S*_ for the degree of content after the treatment. Also, the weight *t*_1_ may be seen as the sum of 7 weights, *t*_*E*_, *t*_*D*_, *t*_*L*_, *t*_*M*_, *t*_*H*_, *t*_*F*_, *t*_*R*_, connected with the importance given to the increase of the thickness of the epidermis, dermis, decreasing of low echogenity pixels, increasing of medium and high echogenity pixels and increasing of ratio LEPi/LEPu, respectively decreasing of the adverse reactions. We change the notation of *t*_3_ with *t*_*C*_.

## 3. Comparing the Results at a General Level and Constructing the MEI Index

In this case, the mathematical model of our medical problem is the following multicriterial optimization problem with the objective function *F* = (*f*_1_,…, *f*_60_) : {0,1}^2^ → **R** ^60^, previously given, and the set of possible solutions *X* = {(*x*_1_, *x*_2_) ∈ {0,1}^2^ | *x*_1_ + *x*_2_ = 1}.

In order to solve this problem, we apply the weight method and we construct the synthesis function Λ : {0,1}^2^ → *R*, Λ(*x*_1_, *x*_2_) = ∑_*i*=1_^60^ *λ*_*i*_ *f*_*i*_(*x*_1_, *x*_2_), for every *x* = (*x*_1_, *x*_2_) ∈ {0,1}^2^, where *λ*_*i*_ is the importance (weight) given to the criteria generated by function *f*_*i*_. Taking into account the previous considerations, we set *λ*_1_ = ⋯ = *λ*_6_ = *t*_*E*_, *λ*_7_ = ⋯ = *λ*_12_ = *t*_*D*_, *λ*_13_ = ⋯ = *λ*_18_ = *t*_*L*_, *λ*_19_ = ⋯ = *λ*_24_ = *t*_*M*_, *λ*_25_ = ⋯ = *λ*_30_ = *t*_*H*_, *λ*_31_ = ⋯ = *λ*_36_ = *t*_*F*_, *λ*_37_ = ⋯ = *λ*_42_ = *t*_*R*_, *λ*_43_ = ⋯ = *λ*_48_ = *t*_*Q*_, *λ*_49_ = ⋯ = *λ*_54_ = *t*_*S*_, *λ*_55_ = ⋯ = *λ*_60_ = *t*_*C*_.

Any optimum solution of the problem

(3) Λ(x1,x2)→max⁡, (x  1  ,x2)∈X

is a max-efficient point (max-Pareto point), so is acceptable. Due to the fact that condition *x*_1_ + *x*_2_ = 1 implies *x*_1_ = 1 − *x*_2_, the solving of problem (3) reduces to the solving of the following problem:

(4) $$\begin{array}{c} \psi \left(u\right)=\overset{60}{\underset{i=1}{\sum }}\lambda_{i}f_{i}\left(u,1-u\right)\to \max,\;u\in \left\{0,1\right\} \end{array}.$$

The simple form of the restrictions conduces immediately to the conclusion: if *ψ*(1) > *ψ*(0), then *u* = 1 is the optimal solution of problem (4); if not, *u* = 0 is the optimal solution. This mathematical result permits us to introduce the MEI index, called medicoeconomic index, attached to the protocol *T*_*i*_, by

(5) $$\begin{array}{cc} \text{ME}{\text{I}}_{T_{i}} & =\overset{3}{\underset{j=1}{\sum }}\overset{2}{\underset{h=1}{\sum }}[p_{ijh}(t_{E}\frac{E_{ijh2}}{E_{ijh1}}+t_{D}\frac{D_{ijh2}}{D_{ijh1}}+t_{L}\frac{L_{ijh2}}{L_{ijh1}}+t_{M}\frac{M_{ijh2}}{M_{ijh1}}\\ & \;\;\;\;\;\;+\;t_{H}\frac{H_{ijh2}}{H_{ijh1}}+t_{F}\frac{F_{ijh2}}{F_{ijh1}})-t_{R}\frac{R_{ijk}}{p_{ijk}}]\\ & \;+\overset{3}{\underset{j=1}{\sum }}\overset{2}{\underset{h=1}{\sum }}\left[p_{ijh}\left(t_{Q}\frac{Q_{ijh2}}{Q_{ijh1}}+t_{S}\frac{S_{ijh2}}{S_{ijh1}}-t_{C}\frac{C_{ijh}}{K}\right)\right]. \end{array}\;$$

In case of small or homogenous samples, we may use the easier formula:

(6) $$\begin{array}{cc} \text{ME}{\text{I}}_{T_{i}} & =\;\overset{3}{\underset{j=1}{\sum }}\overset{2}{\underset{h=1}{\sum }}(t_{E}\frac{E_{ijh2}}{E_{ijh1}}+t_{D}\frac{D_{ijh2}}{D_{ijh1}}+t_{L}\frac{L_{ijh2}}{L_{ijh1}}\\ & \;\;\;\;\;+\;t_{M}\frac{M_{ijh2}}{M_{ijh1}}+t_{H}\frac{H_{ijh2}}{H_{ijh1}}+t_{F}\frac{F_{ijh2}}{F_{ijh1}}-t_{R}R_{ijh})\\ & \;+\overset{3}{\underset{j=1}{\sum }}\overset{2}{\underset{h=1}{\sum }}\left(t_{Q}\frac{Q_{ijh2}}{Q_{ijh1}}+t_{S}\frac{S_{ijh2}}{S_{ijh1}}-t_{C}\frac{C_{ijh}}{K}\right). \end{array}$$

**Remark 2**

Since for the construction of this index we consider only data related to the patient's condition before and after treatment and data referring only this treatment, the index can be used to compare several treatments, unlike ICER and NB that allow comparison of only two treatments and of only two criteria related to cost and effectiveness.

## 4. Special Cases

If we want to compare the overall effectiveness of treatment for two different age groups, we may follow the same steps, except that we consider only the data relative to an age group.

For example, if we want to compare the efficiency of the treatment for each age group, i.e. 40–49, 50–59 and over 60, we construct the MEI index by taking

(7) $$\begin{array}{cc} \text{ME}{\text{I}}_{\text{grup}\;j} & =\overset{2}{\underset{h=1}{\sum }}[P_{1jh}(t_{E}\frac{E_{1jh2}}{E_{1jh1}}+t_{D}\frac{D_{1jh2}}{D_{1jh1}}+t_{L}\frac{L_{1jh2}}{L_{1jh1}}\\ & \;\;\;\;\;+t_{M}\frac{M_{1jh2}}{M_{1jh1}}+t_{H}\frac{H_{1jh2}}{H_{1jh1}})-t_{R}\frac{R_{1jh}}{P_{1jh}}]\\ & \;+\overset{2}{\underset{h=1}{\sum }}\left[P_{1jh}\left(t_{F}\frac{F_{1jh2}}{F_{1jh1}}+t_{Q}\frac{Q_{1jh2}}{Q_{1jh1}}+t_{S}\frac{S_{1jh2}}{S_{1jh1}}-t_{C}\frac{C_{1jh}}{K}\right)\right]. \end{array}$$

In case of small or homogenous samples, an easier formula for computing the MEI index is the following:

(8) MEIgrup  j=∑h=12(tEE1jh2E1jh1+tDD1jh2D1jh1+tLL1jh2L1jh1+tMM1jh2M1jh1   +tHH1jh2H1jh1+tFF1jh2F1jh1−tRR1jh) +∑h=12(tQQ1jh2Q1jh1+tSS1jh2S1jh1−tCC1jhK).

Another special case may be the one connected with the phototype. In this case, we have

(9) $$\begin{array}{cc} \text{ME}{\text{I}}_{\text{phenotip}\;h} & =\overset{3}{\underset{j=1}{\sum }}[P_{1jh}(t_{E}\frac{E_{1jh2}}{E_{1jh1}}+t_{D}\frac{D_{1jh2}}{D_{1jh1}}+t_{L}\frac{L_{1jh2}}{L_{1jh1}}\\ & \;\;\;\;\;+t_{M}\frac{M_{1jh2}}{M_{1jh1}}+t_{H}\frac{H_{1jh2}}{H_{1jh1}})-t_{R}\frac{R_{1jh}}{P_{1jh}}]\\ & \;+\overset{3}{\underset{j=1}{\sum }}[P_{1jh}(t_{F}\frac{F_{1jh2}}{F_{1jh1}}+t_{Q}\frac{Q_{1jh2}}{Q_{1jh1}}\\ & \;\;\;\;\;\;+t_{S}\frac{S_{1jh2}}{S_{1jh1}}-t_{C}\frac{C_{1jh}}{K})]. \end{array}$$

Or, the easier formula is as follows:

(10) $$\begin{array}{cc} \text{ME}{\text{I}}_{\text{phenotip}\;h} & =\overset{3}{\underset{j=1}{\sum }}t_{E}\frac{E_{1jh2}}{E_{1jh1}}+t_{D}\frac{D_{1jh2}}{D_{1jh1}}+t_{L}\frac{L_{1jh2}}{L_{1jh1}}\\ & \;+t_{M}\frac{M_{1jh2}}{M_{1jh1}}+t_{H}\frac{H_{1jh2}}{H_{1jh1}}+t_{F}\frac{F_{1jh2}}{F_{1jh1}}\\ & \;\text{+}t_{Q}\frac{Q_{1jh2}}{Q_{1jh1}}+t_{S}\frac{S_{1jh2}}{S_{1jh1}}-t_{C}\frac{C_{1jh}}{K}-t_{r}R_{1jk}. \end{array}$$

Also, if we want a result based both on the age group and phototype, we have

(11) $$\begin{array}{cc} \text{ME}{\text{I}}_{j,h} & =t_{E}\frac{E_{1jh2}}{E_{1jh1}}+t_{D}\frac{D_{1jh2}}{D_{1jh1}}+t_{L}\frac{L_{1jh2}}{L_{1jh1}}\\ & \;+t_{M}\frac{M_{1jh2}}{M_{1jh1}}+t_{H}\frac{H_{1jh2}}{H_{1jh1}}+t_{F}\frac{F_{1jh2}}{F_{1jh1}}t_{Q}\frac{Q_{1jh2}}{Q_{1jh1}}\\ & \;+t_{S}\frac{S_{1jh2}}{S_{1jh1}}-t_{C}\frac{C_{1jh2}}{K}-t_{R}R_{1jh}. \end{array}\;$$

## 5. Application of the P63 Antiaging Treatment

We exemplify the application of this index for the characterization of the treatment based on P63 antiaging treatment (a metabolic dynamiser composed of alpha hydroxyacids, retinoids, a biomimetic peptidic complex, and gluconolactone incapsulated in liposomes), in the particular case of data presented in the following table and taken from [14]. Our mathematical model was applied on a sample of 30 patients with ages between 40 and 75 years old. Due to our purpose, we divided them in three age interval groups: the first group included patients aged 40–50 years, the second group included the aged ones 50–60, and the last group contained those over 60. All patients included in the study belonged to phototype class II or III, and, therefore, following this criteria, we grouped them into two classes, denoted by 2 and 3. The treatment cost equals 250  monetary units (m.u); the *K* constant is taken equal to 400. For those patients who did not follow the treatment, the cost was considered 100 m.u.

The medical data concerning the group treated with P63 antiaging treatment, as well as the placebo group, are given in the following tables.

In order to compute the MEI index in case of P63 antiaging complex treatment, and placebo one, formula (6) was used. It is important to mention that no side effects were noticed.

We considered equally the weights given for the age groups. We take *t*_*E*_ = 1/8, *t*_*D*_ = 1/8, *t*_*F*_ = 1/2, *t*_*R*_ = *t*_*Q*_ = *t*_*S*_ = 0, and *t*_*C*_ = 1/4. Under these circumstances and using the data from Table 1, the MEI index for the treatment with P63 antiaging complex was *T*_*P*63  complex_ = 0.812143. Using the data of Table 4 to build the MEI index for the placebo treatment, we obtained *T*_0_ = 0.569481. This means that the treatment is worth doing.

Using the same weights, the data from Table 2 and denoting by “Mean” the mean value, b.t. = before treatment and a.t. = after treatment and computing MEI for every age group, we also got the following results: MEI_40–49_ = 0.724915, MEI_50–59_ = 0.897944, and MEI_60–75_ = 0.745013.

The highest value was obtained for the 50–59 group, then for the group 60–75 and then for 40–49 group. It means that the age group 50–59 had the best response to the treatment.

Using the same weights, the data from Table 3 and computing MEI, according with the formula for phototypes 2 and 3, we get MEI_2_ = 0.782524, respectively, MEI_3_ = 0.84888. The greatest value is obtained for phototype 3. This result indicates that the treatment was best perceived for phototype 3.

**Remark 3**

We mention that a change in the weights leads to a change of the index.

## 6. Conclusions

As mentioned before, starting with effective data concerning the treatment with P63 complex, we studied the possibility to quantify the outcome of antiaging skin treatment, from multiple points of view: medical parameters, side effects, cost, convenience of treatment application, the way in which the patient perceive the treatment.

Using multicriteria optimization, we introduced a medicoeconomic index (MEI) capable to perform the required quantification. In its construction, only data connected with a specific treatment were used; therefore, it has the advantage of being able to compare more than 2 treatments.

Further, we gave an application in which we computed the MEI index, in order to compare the P63 antiaging complex treatment with a placebo one (which provided only hydration of the skin). We also constructed specific indexes, according to age groups. This permitted us to compare the effects of P63 complex treatment for different age groups and to establish, from this point of view, a hierarchy of these groups. The same constructions and comparisons were made for the groups of phototypes 2 and 3.

The application presented documents the importance and the facility of use of our index.

## Figures and Tables

**Table 1:** Group treated with P63 antiaging treatment.

Parameters	Mean value before treatment	Mean value after treatment
Thickness of epidermis (mm)	0.117	0.135
Thickness of dermis (mm)	1.537	1.719
LEPs/LEPi	1.149	1.574

**Table 2:** Group treated with P63 complex, based on age groups.

Parameters	Mean b.t. 40–49	Mean a.t. 40–49	Mean b.t. 50–59	Mean a.t. 50–59	Mean b.t. 60–75	Mean a.t. 60–75
Thickness of epidermis (mm)	0.114	0.135333	0.11675	0.1345	0.12025	0.136125
Thickness of dermis (mm)	1.8585	1.901	1.386313	1.740563	1.367625	1.506625
LEPs/LEPi	1.204593	1.457353	1.096062	1.398047	1.213842	1.510156

**Table 3:** Group treated with P63 antiaging treatment, based on phototype.

Parameters	Mean b.t. phototype 2	Mean a.t. phototype 2	Mean b.t. phototype 3	Mean a.t. phototype 3
Thickness of epidermis (mm)	0.114625	0.129938	0.12	0.141
Thickness of dermis (mm)	1.605313	1.768063	1.460571	1.644214
LEPs/LEPi	1.198661	1.580802	1.092623	1.567998

**Table 4:** Placebo group.

Parameters	Mean value before treatment	Mean value after treatment
Thickness of epidermis (mm)	0.1358	0.13505
Thickness of dermis (mm)	1.49635	1.57155
LEPs/LEPi	0.924757	1.042922
